# Case example of a jail-based cancer prevention clinical trial: Social determinants of health framework, novel experimental design, and retention strategies to facilitate long-term follow-up of clinical trial participants

**DOI:** 10.1017/cts.2023.561

**Published:** 2023-06-23

**Authors:** Pablo Kennedy, Rubina Ratnaparkhi, Jaehoon Lee, Jason E. Glenn, Patricia J. Kelly, Kim S. Kimminau, Stephanie Assimonye, Megha Ramaswamy

**Affiliations:** 1 University of Kansas School of Medicine, Kansas City, KS, USA; 2 Texas Tech University, Lubbock, TX, USA; 3 University of Missouri Kansas City, Kansas City, MO, USA

**Keywords:** Jail, clinical trial, social determinants of health, cancer prevention

## Abstract

Clinical trials conducted with incarcerated populations are rare. We present a case example of one such jail-based cancer prevention clinical trial to demonstrate the importance of including a theory-driven approach to intervention framing, novel experimental designs to boost access to low-risk trials, and retention strategies for long-term follow-up of hard-to-reach populations. As such we offer a social determinant of health framework to ensure cancer prevention research is conducted through the lenses of health promotion and health equity. Deviations from the gold-standard randomized control design, transparent systematic allotment, and street-based outreach retention strategies contribute to the feasibility of conducting clinical trials in carceral settings and after people leave jail. Best practices presented can be used in design and conduct of future clinical trials with criminal legal system-involved populations.

## Introduction

Incarcerated populations once constituted most biomedical research subjects [1]. This research was largely exploitative for benefit of nonincarcerated populations. In the 1970s, federal reforms mandated that research on people experiencing incarceration be permitted only when conducted for their specific benefit. Over 9% of the U.S. population has criminal legal system involvement, and poor Black and Latinx patients are disproportionately affected [2]. This population faces poorer health outcomes due to intersectional effects of structural racism, social determinants of health, and lesser access and limited generalizability of cutting-edge biomedical research trials [3,4].

From 2008 to 2012, less than 0.1% of NIH-awarded grants were allocated for research with people experiencing incarceration [5]. Of 3,113 published clinical trials, only 95 focused on incarcerated population, only 13 were specific to incarcerated women [6], and only four studies included up to two years of longitudinal follow-up [6]. Over half of identified studies have a risk of bias that is high or unable to be determined [7]. Sources of bias included issues around randomization, blinding, and protocol deviations with limited transparency on methods. Publication bias is also evident in the large proportion of studies reporting only positive results. This highlights significant ongoing challenges in implementation of high-quality research in the jail setting. A recent review concluded that randomized controlled trials (RCTs) are feasible in prisons and proposed theoretical best practices including using pilot studies to test ideas, building effective working relationships with prison staff, and conducting iterative process evaluations [7]. However, limited specific implementation data exist on how to enact best practice protocols.

In 2010, the World Health Organization (WHO) Commission on Social Determinants of Health (SDOH) proposed a single, unifying conceptual framework to more accurately characterize cause-and-effect relationships from a health equity and social justice lens [8]. The framework outlines that in a given context (social and political mechanisms establishing social hierarchies), structural determinants create stratification among individuals in terms of access to resources and agency and differential exposure to vulnerabilities and health-damaging conditions [8]. Structural determinants operate through intermediary determinants including material or psychosocial circumstances, behaviors, and/or biological factors and/or health system design to influence health outcomes [8]. The SDOH framework intentionally guides policy directions by clarifying mechanisms of causation for ongoing health inequities to avoid conflation and subsequent ineffective mismatching of interventions to the SDOH they are intended to address. In the past decade, few studies assessed study outcomes with a priori investigation of structural and intermediary determinants, limiting reproducibility and generalizability of results.

Women represent one of the fastest-growing segments of incarcerated persons with a 750% increase in incarceration rates between 1980 and 2017 [9]. Incarcerated women carry a higher burden of exposures to health-compromising conditions including poverty, low education, physical and sexual trauma, tobacco use, and sexually transmitted infections [10]. This cumulative risk exposure burden is seen as a 4–5x greater prevalence of cervical cancer in incarcerated women compared to age-matched samples of noninstitutionalized women [4]. This disparity has persisted for nearly 50 years [4,11]. Correlates of screening and follow-up include low cervical health literacy, living in states without Medicaid expansion (in a three-city comparison), not having a primary care doctor, and lack of specific instructions about follow-up [11–14].

Ramaswamy *et al* (2017) developed the Sexual Health Empowerment (SHE) Project’s cervical health literacy intervention to increase incarcerated women’s knowledge about cervical health, accuracy of beliefs regarding cervical cancer, and self-efficacy and confidence in navigating the healthcare system to obtain cervical cancer screening and follow-up [15]. We previously demonstrated significant immediate improvement in cervical health literacy domains [15]. Follow-up was conducted for participants who transitioned back to their communities over three years. Here, we present long-term outcomes of the SHE Project. We hypothesized that the SHE Project would promote durable increases in cervical health literacy associated with increased overall uptake of cervical cancer screening. Having completed the extended follow-up period, we now report how WHO’s SDOH model was employed to operationalize implementation and study design. We offer generalizable insights into how trials can be conducted to include individuals experiencing and leaving incarceration to promote health equity.

## Methods

### Background on Sexual Health Empowerment (SHE) Project intervention/site

The SHE Project was developed as a cervical health literacy intervention delivered in a small-group (5–10) format over two-hour periods on five sequential days. Content was designed to improve cervical health knowledge base, accuracy of beliefs around screening and treatment, self-efficacy in screening and follow-up, and confidence in navigating the healthcare system. Sessions were interactive and included reflection on women’s individual experiences navigating their social and political contexts including specific knowledge and capital strengths and gaps. This included emphasis on the context of romantic and sexual relationships, family, community, intersectionality of race-, class-, and gender-specific health outcomes, and rejecting assumptions about how women should take care of their health [16]. The content was informed by prior qualitative data and needs assessment we had collected in a similar local population [17], which aligns with a known best practice for research with criminal legal involved populations – our research centered on the specific needs and educational, social, and cultural backgrounds of this population and built on past relationships between the study team and the population [18]. A needs assessment highlighted womens’ desire to engage with community social relations, local resources, and in-group knowledge [17]. As a result, the group-based intervention emphasized “social transformation” and identified strategies women could use to navigate criminal justice involvement, community reentry, interpersonal relationship, and health services inside and outside of jail. We aimed to emphasize empowerment by increasing knowledge and building self-efficacy by openly discussing challenges and highlighting participant-driven solutions to navigating healthcare. More detail was previously reported [15,19].

We also conducted a feasibility pilot study of the intervention and conducted an extensive retrospective process evaluation prior to undertaking the present longitudinal study [17]. This was conducted over one week with seven participants. This demonstrated that participants enjoyed and looked forward to the program, learned new information, and would participate again. Recommendations included making the program longer in duration and revising some confusing survey language (which was completed). The retrospective process evaluation was invaluable in ensuring feasibility of the present study. We assessed five questions regarding feasibility: (a) Were we able to recruit participants? (b) Were we able to retain participants? (c) Was the jail amenable to our implementation? and (d) Did jail administrators facilitate intervention delivery and follow-up of participants? [17]. We used five questions to assess the intervention: (a) Did the needs assessment data inform the intervention? (b) Did the theoretical framework inform the intervention? (c) Was the intervention delivered as designed? (d) What was the quality of intervention delivery? and (e) Were the instruments selected appropriate for the intervention? [17]. Examination of the needs assessment data highlighted multiple similarities and differences for our specific population from previously published literature; addressing these areas of alignment and contrast allowed us to design evidence-based content for the intervention tailored to their needs [17].

The intervention was conducted at three minimum- and medium-security county jails in the Kansas City metropolitan area. Two jails were urban with 300–800 inmates, and one was suburban with 1000 inmates, of which 15% were female. All inmates undergo medical intake and pay fees for requested treatment and/or medicines through jail health services provided by contracted corporations. Preventive healthcare services (e.g., Pap test, STI screening) were available only as medically necessary.

Participants were recruited on a rolling basis if eligible (i.e., if present at one of the three study sites, age 18 or above, able to understand and speak English). Participants were excluded if displaying disruptive behaviors such that they were not able to participate in informed consent and the intervention. This sample has been previously described [15].

### Variables, Outcome Measures, and the WHO SDOH Framework

All participants completed a 158-item baseline survey. Participants in the immediate intervention group completed an 82-item postintervention survey upon program completion. Participants in the waitlist control group completed a 73-item preintervention survey before beginning the program and the 82-item postintervention survey upon program completion. Thus, changes in outcomes and covariates were assessed using changes in response from baseline to postintervention survey for the immediate intervention group and from baseline to preintervention survey for the waitlist control group to approximate “true” control conditions. At three-year follow-up, all participants, irrespective of initial randomization group, completed the same 82-item postintervention survey. Thus, changes in outcomes between three-year follow-up and baseline were assessed in aggregate among the entire study population; by the time of assessment at long-term follow-up, all study participants had participated in the literacy intervention and a “true” control group no longer existed.

**Figure 1.**
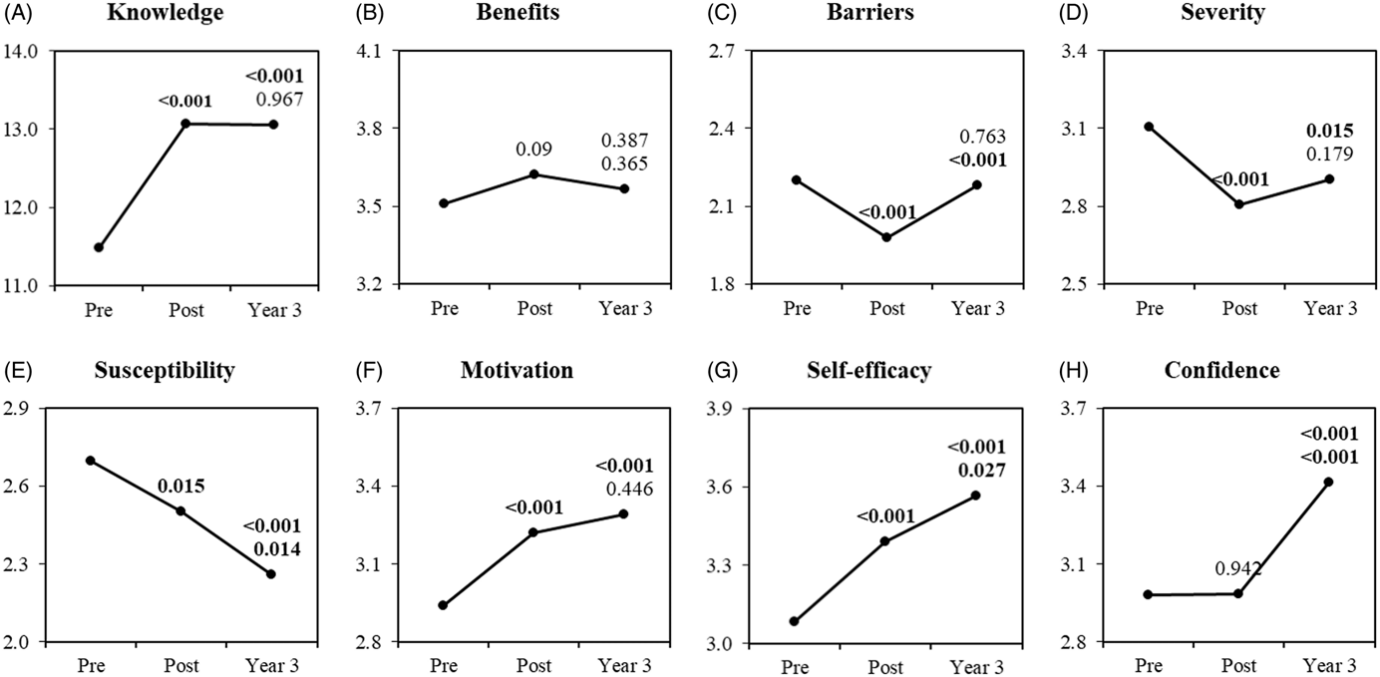
Women’s health literacy scores preintervention, postintervention, and at year 3 (n = 111). *Note.* the value at the “post” point is the p-value of test for comparing preintervention vs. postintervention; the upper and lower values at the “year 3” point are the p-values of test for comparing preintervention vs. year 3 and comparing postintervention vs. year 3, respectively. p values less than 0.05 are in boldface.

The primary study outcome was change in cervical health literacy. This was operationalized as changes in sub-scores for eight domains of cervical health literacy between preintervention and postintervention surveys (Fig. 1). Cervical health knowledge (1A) was assessed with the Pap Knowledge Scale [20]. The Health Belief Model Scale for cervical cancer and Pap smear test [21] was used to assess perceived benefits of screening (1B), barriers to screening (1C), seriousness of cervical cancer (1D), susceptibility to cervical cancer (1E), and motivation for screening (1F). Self-efficacy (1G) was assessed using the Self-Efficacy Scale for Pap smear screening participation [22], and confidence (1H) around cervical health screening and follow-up was assessed with three questions designed by our group [15]. Criteria that informed our choice of instruments included ability to measure domains of interest, prior validation from the literature, prior use in vulnerable populations, and instrument length [17].

We intentionally chose to define cervical health literacy comprehensively including not only knowledge, but also the beliefs, confidence, and self-efficacy required to effectively navigate health systems for cervical health promotion [15]. A prior qualitative study with women experiencing incarceration showed that despite Short Test of Functional Health Literacy in Adults scores in the “adequate” range, women had significant gaps in functional cervical health literacy [13]. Notable gaps included misunderstandings about the purpose of a Pap test, belief in the utility and importance of Pap tests, causes of cervical cancer, and how cervical cancer prevention works [13]. These misunderstandings contributed to fear and ambivalence about cervical cancer screening and, along with concerns in navigating the stigma of criminal-legal involvement in the healthcare system, created unique challenges in self-efficacy and women’s ability to act on knowledge for cervical cancer prevention [13]. Their knowledge, beliefs, and self-efficacy around cervical cancer screening are also impacted by long trauma histories, mental health problems, trading sex for money, drugs, or shelter, drug use, and cycling in and out of the carceral system [13]. In 2004, the Institute of Medicine (IOM) published a report entitled (*Health Literacy, A Prescription to End Confusion*) noting “Health literacy level is the product of a complex set of skills and interactions on the part of the individual, the healthcare system, the education system, and the cultural and societal context [23].” Our method of operationalizing cervical health literacy reflected findings of the IOM report and fulfilled a critical need to tailor the intervention to address the group’s unique risks within their environmental, systemic, and societal context [13].

**Figure 2.**
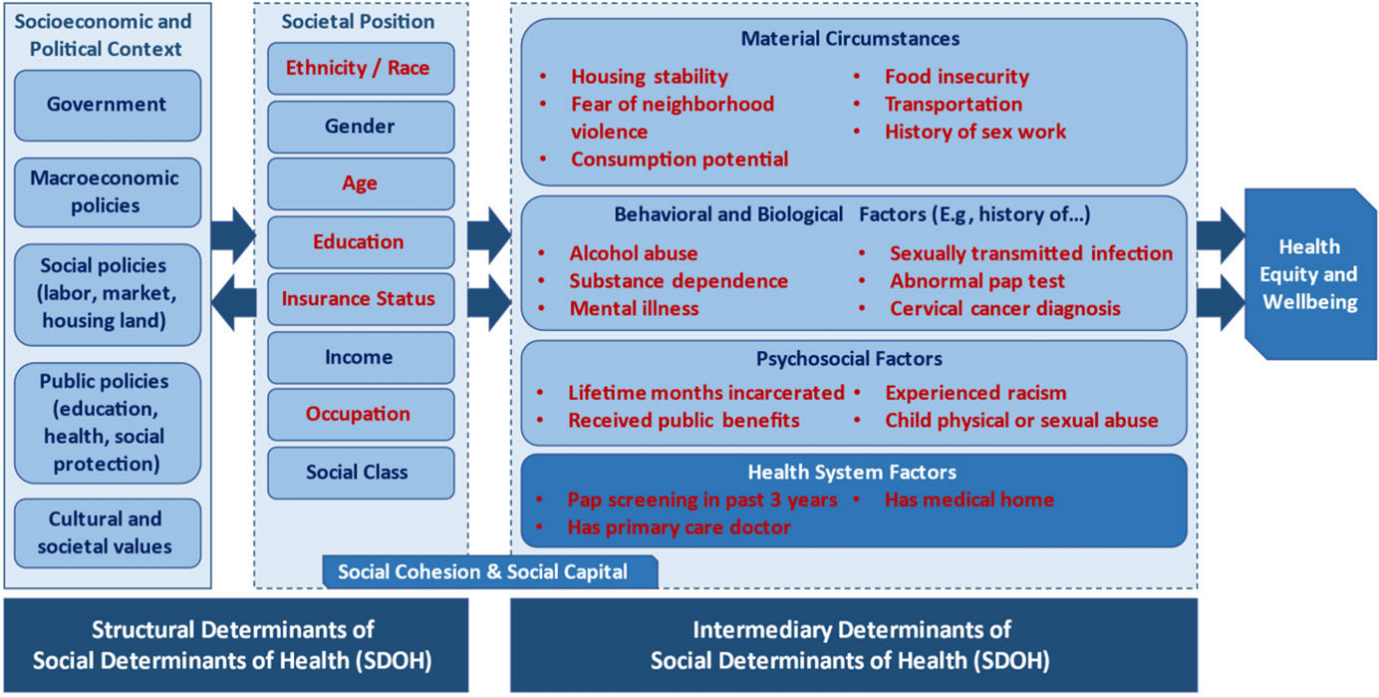
WHO social determinants of health (SDOH) conceptual framework with relevant study variables and covariates mapped (red text) to corresponding domain.

Two secondary outcomes were also investigated. Rates of being up-to-date with cervical cancer screening were assessed at preintervention and postintervention assessments by asking participants if they have had a Pap test completed within the past three years. Of note, a prior study demonstrated self-report to be an accurate measure of cervical cancer screening rates among incarcerated persons [24]. We also assessed the overall attrition rate among the study cohort. We compared participants who completed three-year follow-up with those who were lost to follow-up to determine if there were differences in reported structural and/or intermediary social determinants of health that may impact incarcerated persons’ ability to participate in a longitudinal clinical trial. Figure 2 demonstrates how survey items relate to the structural and intermediary determinants within WHO’s SDOH framework, which reinforced classification of primary/secondary outcomes (dependent variables) and defined covariates for analysis.

### Statistical analysis

Participants’ cervical health literacy was summarized based on calculated scores at three-time points – preintervention, immediate postintervention, and 36 months postintervention for each of the contributing subscales. Student’s *t*-test was performed to examine changes in each variable over the 3-year period. The prevalence of up-to-date Pap test screening was calculated by the same method. There was no paired analysis of variables at three-year follow-up given absence of a true control group.

Descriptive statistics were utilized to summarize sociodemographic factors and measured structural and intermediary SDOH. Bivariate tests (independent-samples *t*- test, Chi-square, or Fisher’s exact test as appropriate) were conducted to compare differences in measured SDOH between participants retained for follow-up for the full duration of the trial (*n* = 111) versus those who lost to follow-up (*n* = 71) after intervention completion. In line with an intent-to-treat approach, when there were missing observations due to either attrition or nonresponse, all available data from partial measurements were included in the analysis. All analyses were conducted using SAS 9.4 (SAS Institute, 2002–2012). Statistical significance was determined at 0.05 α level.

### Study design, jail recruitment, and retention considerations

A randomized controlled trial (RCT) is considered the gold standard to determine an intervention’s efficacy [6]. RCT design permits valid causal inference on the impact of an intervention on a set of predefined outcomes in a population relative to a control group of individuals from the same population who receive either a previously accepted gold standard or no intervention [6]. WHO’s SDOH model acknowledges that incarcerated women occupy severely disadvantaged social positions associated with persistent disparities in cervical health due to lesser access to resources and increased exposure to risk factors [8,12]. In this context, we felt it would be unethical to only selectively offer a low-risk intervention (provision of health information) anticipated to provide at least some benefit and minimal harm.

There are several justifications for this determination. To date, a “gold standard” intervention to improve cervical health literacy has yet to be defined.13 Many sources of varying quality developed educational materials on women’s health, but few were validated for use among vulnerable populations.13 Some authors propose possible negative ethical ramifications about cervical cancer education for incarcerated populations associated with increasing awareness of cervical cancer risk while participants have inadequate access to resources to address this risk [25]. In designing intervention materials, cervical cancer was characterized as a low absolute risk and with good odds of prevention and treatment with early detection.15 This counters prior educational approaches that aimed to intensify perceptions of risk and cervical cancer severity to promote greater screening uptake.26 Our data confirmed the intervention diminished participants’ fears of cervical cancer. Moreover, fear and lack of understanding regarding abnormal screening results and risk of cancer are prevalent and contribute to delays and incomplete follow-up among nonincarcerated women including those with adequate resources for follow-up [12,26]. It would be unethical to withhold educational resources from a vulnerable population for fear that exist broadly in the general population. This approach shifts the paradigm in correctional health from reactive medical care to proactive health promotion with the goal of providing incarcerated individuals with knowledge, skills, and referrals to maintain health both while incarcerated and after release [27].

Thus, we developed a quasi-experimental design to test intervention efficacy while ensuring all participants received the intervention. Participants were systematically assigned such that half of each recruitment cohort received the intervention in week one and the other half, or “waitlist control” group, received the intervention in week two. This was completed by alternating assignment based on patient seating during the informed consent process. Deviations only occurred if/when a recruitment cohort contained fewer than five participants who then were all assigned to the intervention group. Though this practice was associated with a slight imbalance in study group sizes (54.4% intervention cases and 45.6% control cases), there were no significant differences in structural and intermediary SDOH and health history factors except greater prevalence of abnormal cervical cancer screening history in the waitlist control group.

We observed that use of a transparent assignment strategy helped participants to trust the study steam and fostered a sense of fairness and agency among participants. Nevertheless, some in the waitlist control group expressed disappointment due to possibility of release prior to the intervention. In these instances, a standardized script was reviewed emphasizing the importance of following “valid science rules” to ensure fairness and accurate assessment of program effectiveness. This explanation resonated with some participants and frustrated others, but it was our attempt to convey the rationale. We were able to meet recruiting goals despite this limitation.

Multiple factors facilitated trial recruitment and enrollment. We used social networks and cold calls to gain access to jails. In one case, a graduate student was a former state senator and introduced us to a jail sheriff; in another case, a department administrator married to a jail deputy made an introduction; in a third case, we cold-called a jail asking to meet. We brought breakfast to prepandemic in-person meetings, introduced our team, and stated our goals. We worked with jail administrators to develop plans for recruitment, intervention/survey administration, and participant payment.

A multimodal recruitment campaign was used including flyers (which said “Are you interested in participating in a study about your sexual health?”) [28], word-of-mouth, and direct discussions with study staff. Every week preceding participant recruitment, three members of the team would go into the housing unit and talk to those incarcerated about the study and education programs. By going in person and showing that we were kind human beings, word traveled fast among the potential participants that what we were offering was low risk and a respite from confinement in housing units for sessions. The study staff all had prior experience in working and conducting research with incarcerated persons, and ethnoracially matched personnel were among the study team. We also utilized a training manual to prepare staff and debriefed weekly about experiences and feelings.

We administered surveys and the intervention in small program rooms or libraries with groups of 6–10 people per session that had registered one day prior. The intervention was delivered over a 5-day session in one week based on prior research to account for rapid turnover in jails [28]. The intervention was made to be two hours in length from 10 a.m. to 12 p.m. to accommodate the jail’s meals and court visits. We arranged tables so that participants could sit in a circle, with three of our staff members interspersed to attempt to minimize existing power imbalances (given staff could move freely between jail and community, whereas participants could not). We read through consent documents and surveys aloud along with participants to increase accessibility. We went over intervention content (largely group discussion based), using the same seating arrangements. Grounding techniques (e.g., mindfulness, controlled breathing, guided visualization) were utilized if issues of trauma came up [19]. We were also prepared to refer to mental health services at the jail if needed.

Jails are short-term facilities (people waiting for trial, sentencing, or sentenced to one year or less), so the parent study was designed to follow people after jail to better understand community-based cancer prevention practices. Thus, retention after follow-up over a three-year study period was critical. Wickliffe *et al* (2019) reviewed retention strategies undertaken in this study [29]. Emphasizing a “no judgment zone” and confidentiality during sessions and icebreaker activities fostered a safe space for participants. Obtaining multiple forms of contact information including three persons who “no matter what, know where you are,” frequented businesses and community organizations, and names on social media accounts facilitated follow-up. Routine searches in public databases and networking within a private study social media group also supported enduring connections. Of note, communications included messaging beyond follow-up reminders including personalized letters, newsletters, birthday, and holiday cards including prepaid postage to enable responses. Telephone often was a less reliable mode of reaching participants, though designating a study cell phone helped participants reach the research team. When designing a trial for incarcerated persons including long-term follow-up after release, it is critical to plan adequate time and budget for incentives, robust communication and outreach, and a savvy experienced research staff.

## Results

### Cervical cancer screening and long-term cervical health literacy outcomes

**Figure 3.**
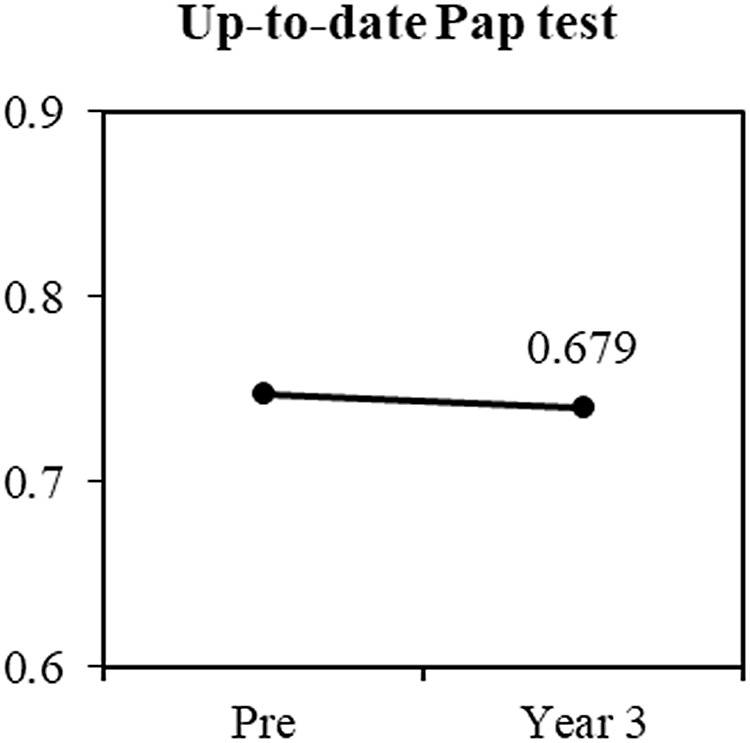
Proportion of up-to-date Pap screening preintervention (n = 261) and at year 3 (n = 111). *Note.* the value at the “year 3” point is the *p*-value of test for comparing preintervention vs. year 3. *p* values less than 0.05 are in boldface.

Table 1 presents demographic characteristics of study participants. Rates of up-to-date cervical cancer screening were high throughout the study period; 74% of participants reported having a Pap test within 3 years in the preintervention phase, and 75% reported the same at three-year follow-up (Fig. 3)

**Table 1.** WHO based determinants of health for those who were retained at year 3 versus those who were lost to follow-up

	Total (*n* = 182)	Retained at year 3 (*n* = 111)	Lost to follow-up (*n* = 71)	*p* ^a^
**Structural determinants**				
Age	33.84 ± 9.48	34.07 ± 10.04	33.47 ± 8.57	0.673
Race				0.053
*White*	92 (50.5 %)	52 (46.8 %)	40 (56.3 %)	
*Black*	53 (29.1 %)	36 (32.4 %)	17 (23.9 %)	
*Other*	21 (11.5 %)	14 (12.6 %)	7 (9.9 %)	
*Multiple*	12 (6.6 %)	9 (8.1 %)	3 (4.2 %)	
Ethnicity				**0.003**
*Latinx*	17 (9.3 %)	10 (9.0 %)	7 (9.9 %)	
*Non-Latinx*	158 (86.8 %)	101 (91.0 %)	57 (80.3 %)	
Education				0.620
*Less than high school*	62 (34.1 %)	38 (34.2 %)	24 (33.8 %)	
*High school or beyond*	115 (63.2 %)	71 (64.0 %)	44 (62.0 %)	
Employment status				0.501
*Employed*	61 (33.5 %)	37 (33.3 %)	24 (33.8 %)	
*Unemployed*	108 (59.3 %)	68 (61.3 %)	40 (56.3 %)	
**Intermediary determinants**				
**Material circumstances**				
Housing				0.763
*Housing stability*	126 (69.2 %)	79 (71.2 %)	47 (66.2 %)	
*Housing instability*	45 (24.7 %)	26 (23.4 %)	19 (26.8 %)	
Neighborhood				**0.028**
*Fear of violence*	68 (37.4 %)	38 (34.2 %)	30 (42.3 %)	
*No fear of violence*	108 (59.3 %)	72 (64.9 %)	36 (50.7 %)	
Consumption potential				0.204
*Basic needs financial stability*	82 (45.1 %)	50 (45.0 %)	32 (45.1 %)	
*Basic needs financial instability*	91 (50.0 %)	58 (52.3 %)	33 (46.5 %)	
Food insecurity	46 (25.3 %)	21 (18.9 %)	25 (35.2 %)	**0.042**
No reliable source of transportation	52 (28.6 %)	29 (26.1 %)	23 (32.4 %)	0.187
History of exchanging sex	60 (33.0 %)	39 (35.1 %)	21 (29.6 %)	0.479
**Behavioral and biological factors**				
History of harmful alcohol use	94 (51.6 %)	56 (50.5 %)	38 (53.5 %)	0.686
History of substance dependence	112 (61.5 %)	72 (64.9 %)	40 (56.3 %)	0.249
History of mental illness^a^	133 (73.1 %)	82 (73.9 %)	51 (71.8 %)	0.762
History of STIs^b^	115 (63.2 %)	72 (64.9 %)	43 (60.6 %)	0.557
History of abnormal Pap test^c^	95 (52.2 %)	57 (51.4 %)	38 (53.5 %)	**0.046**
Cervical cancer diagnosis	24 (13.2 %)	14 (12.6 %)	10 (14.1 %)	0.897
**Psychosocial factors**				
Lifetime months incarcerated	25.55 ± 47.77	23.51 ± 39.02	28.75 ± 59.12	0.517
Received public benefits	87 (47.8 %)	55 (49.5 %)	32 (45.1 %)	<**0.001**
Experienced racism	70 (38.5 %)	45 (40.5 %)	25 (35.2 %)	0.688
Child physical or sexual abuse	50 (27.5 %)	34 (30.6 %)	16 (22.5 %)	0.094
**Health system factors**				
Pap screening in past three years	124 (68.1 %)	78 (70.3 %)	46 (64.8 %)	0.129
Has primary care doctor	69 (37.9 %)	44 (39.6 %)	25 (35.2 %)	0.521
Has medical home	125 (68.7 %)	80 (72.1 %)	45 (63.4 %)	0.250
No insurance coverage	96 (52.7 %)	55 (49.5 %)	41 (57.7 %)	0.505

*M* ± *SD*; *n* (%).

a *p* values less than 0.05 are in boldface.

b Lifetime diagnosis of depression, anxiety, bipolar disorder, or posttraumatic stress disorder by a clinician.

c Lifetime diagnosis of hepatitis B or C, human immunodeficiency virus, syphilis, gonorrhea, chlamydia, trichomoniasis, herpes, or HPV by a clinician.

At three years follow-up, participants had significant increases in six of eight domains of cervical health literacy (see Fig. 1) including increased knowledge about cervical health (*p* < 0.001), reduced perception of seriousness of cervical cancer (*p* < 0.001), reduced perception of susceptibility to disease (*p* < 0.01), as well as greater motivation (*p* < 0.001), self-efficacy (*p* < 0.001), and confidence (*p* < 0.001) for seeking out cervical health screening and follow-up care. The decrease in perceived barriers to cervical cancer screening noted immediately that postintervention was no longer significant.

### SDOH and trial attrition

Of 283 incarcerated women who expressed interest in the SHE Project, 261 consented, and 184 (70.5%) completed the intervention and immediate postintervention assessment. In total, 111 (42.5%) remained in the trial and completed all follow-ups three years postintervention.

There were no significant differences between retained participants and those lost to follow-up in terms of most structural determinants of health including age, education, and employment (see Table 1). Black and minority participants were less frequently lost to follow-up than white participants, although the difference was only marginally significant (*p* = 0.053). Latinx participants were more likely to be lost to follow-up compared to non-Latinx participants (*p* = 0.003). Among intermediary SDOH, differences in material circumstances were most prominent. Total of 35.2% of patients lost-to-follow-up reported experiencing food insecurity in contrast to only 18.9% of patients who completed the study (*p* = 0.042). Perception of neighborhood violence was also more prevalent among participants lost-to-follow up (42.3%) relative to participants who completed the study (34.2%, *p* = 0.028). Participants lost to follow-up had statistically higher rates of prior abnormal Pap test and lower rates of public benefit use.

## Discussion

Our study showed that a brief, low-technology, easy-to-implement, and jail-based education intervention can increase cervical health literacy among incarcerated women. Implementation of our interventional trial, with a few intentional, a priori, and defensible deviations from the RCT “gold standard,” demonstrates that longitudinal high-quality experimental research can be conducted effectively with incarcerated populations. The vast majority of studies with criminal-legal involved persons are majority or only men and measure outcomes only in prison or only after release [6]. Recent systematic reviews noted that fewer than eight studies followed participants longitudinally with retention rates ranging from 40–80% at one- to two-year follow-up [6,7]. We were able to maintain a retention rate within this range with significantly longer follow-up period in the community, combating prevailing contentions that longitudinal studies including long-term follow-up after release are not feasible.

Our intervention yielded a significant and sustained increase in cervical health literacy over three years. Previously published one-year follow-up data from this cohort demonstrated a corresponding increase in up-to-date status with Pap test screening, but this did not persist at three-year follow-up [30]. Our findings parallel three systematic reviews that yielded mixed evidence with an inconsistent relationship between improved health literacy and increased cervical cancer screening rates and varying correlations between subdomains including knowledge, worry, and perceived barriers across studies [31]. Criminal legal involved women face many challenges in cervical cancer screening and follow-up upon release from jail including inadequate knowledge, unstable life circumstances, competing demands, and financial challenges [12].

While incarcerated, preventive healthcare services, including Pap tests and STI screening, were not available, and all medical treatment and medicines requested required a fee through jail health services. The area is served by local health departments, low-cost clinics, and community-based behavioral health clinics, but there are no navigation or transition services to help connect individuals with these services and ensure continuity of care. Kansas is one of few states that did not pass Medicaid expansion, which limits access to affordable health insurance for many criminal-legal involved individuals. Additionally, in Kansas, Medicaid is canceled during one’s sentence, and women must re-enroll after release, which can create extensive periods of gaps in coverage. In our sample, 52% of patients lacked health insurance. Though two-thirds of participants endorsed having a primary medical home, only just over one-third of participants reported having a primary care doctor, which raises questions about their access to care and ability to navigate the healthcare system. Over one-fifth of our sample also reported housing instability, fear of neighborhood violence, food insecurity, and/or lack of access to transportation, and over half were unemployed. These elements of social insecurity represent tangible barriers to accessing healthcare that make it challenging for criminal-legal involved populations to act on cervical health promotion even with increased cervical health literacy and self-efficacy. Without accompanying interventions to address SDOH, there is a limit to which individual education and behavior change can combat systemic disadvantage.

Our experience demonstrates the importance of incorporating WHO’s SDOH model in studies with incarcerated women from intervention conception to study design and analysis. Our intervention uniquely contextualized cervical health education in its intersectionality with participants’ lived experiences of poverty, trauma, sex work, stigma, and criminal-legal involvement. This represents a departure from most prior interventions more focused on knowledge, statistics, and written health literacy [31]. Feminism was centered in the education intervention including acknowledging and discussing political and social structures that embed gender-based assumptions that affect treatment of women while in jail and impact struggles after release including access to income assistance, child custody, and reproductive choice/healthcare access [19]. The project also emphasized relationships based on feminist cultural-relational theory by leveraging the small-group format to build community and collective consciousness [16]. This gave participants a sense of agency and voice as individual experiences were honored in group discussions [19]. Finally, the SHE project incorporated the feminist concept of embodiment (encouraging participants to talk about their bodies and reclaim their lived experience in their bodies) and by exploring how experiences evolve differently based on intersections with race/nationality, education, income, etc [19]. All study team members were trained in trauma-informed care and had experience in social work and working with criminal justice-involved populations. Sessions included group discussion of readings, personal reflection, and role play. Concepts of cervical knowledge, belief, and self-efficacy were discussed with feminist and social theory lenses reviewing themes of empowerment, stigma, trust, social support, and sharing of local resources [19]. Further detail on how feminism and social theory were embedded in the intervention are reviewed by Emerson et al (2019).

We also used predefined structural and intermediary SDOH to define demographic variables and exploratory covariates for data analysis. We found that two factors within the material circumstances domain of intermediary determinants, food insecurity, and neighborhood environment were disproportionately prevalent among participants unable to complete three-year follow-up in our trial. Intentional data collection around SDOH can identify participants at risk of loss to follow-up to consider offering support, referrals, and/or resources as needed. Knowledge of contributing SDOH factors is also critical to drive appropriate policy work in conjunction with ongoing research, as both research and policy are needed to affect long-term and sustainable change in health equity.

This case study illustrates many of the practical challenges in designing high-quality experimental studies in the jail setting. There were two key deviations from gold standard RCT design: use of transparent systematic assignment of participants rather than blinded randomized allotment and use of a waitlist control group such that analysis at long-term follow-up required prepost comparisons due to lack of preservation of the control group. Though both decisions carry risk of bias as outlined in the methods section, we propose that these limitations do not outweigh the benefit of prospective interventions relative to quasi-experimental retrospective analyses. A prior systematic review of RCTs evaluating participants during imprisonment and year after release noted that 91 of 95 studies had high or unclear risk of bias, none described true random sequence allocation, and few provided information on procedures for allocation concealment, blinding, and management of incomplete and/or missing data.6 Normalizing open reporting of employed contingencies and their underlying rationale is important to be able to evaluate study quality while avoiding perpetuating publication bias and discouraging research groups from working effectively with underserved incarcerated populations [7].

The study also emphasizes the need for comprehensive, multimodal, and intensive outreach to support participant recruitment and retention. Particularly where long-term follow-up of criminal legal involved women is required, persistent, varied, and coordinated outreach efforts and significant time and expense are required [29]. This includes utilizing participants’ social networks, social media, and letters and trying and retrying strategies. Prior studies have confirmed the intensity, expense, and variety required [29,32]. Two study team members led follow-up efforts. It is possible that some gains in confidence and self-efficacy stemmed from relationships with an invested study team and the effect of social support in navigating life after release in addition to the role of the intervention itself. Additionally, there was ethnoracial congruency between researchers delivering the intervention and participants, which also may have contributed to greater retention rates among ethnoracial minorities in our study [33]. This result counters pervasive misconceptions that Black patients are less likely to participate in long-term clinical trials [33,34]. In pursuit of health equity, it is critical to acknowledge that participants facing systemic patterns of disadvantage associated with incarceration and systemic racism naturally may need additional and/or different kinds of support to be able to access and engage with the resource.

Finally, there is the practical reality of no good information about cervical cancer screening in jails. Due to their short-term nature, jails, as opposed to prisons, rarely offer preventive health services, including cancer screening. In a four-state study of 192 Midwestern jails, we found that only 3% offered cervical cancer screening [29]. So little information is available about cancer screening services in jails or prisons, which represents missed opportunities in service and research.

The strengths of our study lie in the intervention grounded in social and feminist theory and the tailored, comprehensive trial design, and retention strategies to meet participants where they are. An additional strength is the open and detailed description of study procedures to increase reproducibility, decrease uncertainty around risk of bias, and increase access to innovative study design elements. Limitations of the study stem from the same factors i.e., departure from gold standard RCT design, small sample size with attrition despite retention strategies, and the outstanding question of whether our approach can be scaled and implemented in other areas. Finally, with an intention-to-treat analytical approach, partial sets of outcome measurements were not lost but analyzed in the analysis. Further studies are required to assess if our intervention can improve cervical health literacy in other groups of incarcerated women.

## Conclusions

The SHE Project improved cervical health literacy among incarcerated women with a durable effect up to three years after the intervention, though this was not accompanied by sustained improvement in cervical cancer screening rates between year 1 and year 3 in the context of high baseline screening rates. Novel clinical trial designs can be implemented with comprehensive reporting to support risk of bias assessment while increasing feasibility of conducting meaningful research in the carceral setting. Addressing SDOH in study design and analysis can increase promotion of health equity in studies with vulnerable populations.
